# Characterization of the plant homeodomain (PHD) reader family for their histone tail interactions

**DOI:** 10.1186/s13072-020-0328-z

**Published:** 2020-01-24

**Authors:** Kanishk Jain, Caroline S. Fraser, Matthew R. Marunde, Madison M. Parker, Cari Sagum, Jonathan M. Burg, Nathan Hall, Irina K. Popova, Keli L. Rodriguez, Anup Vaidya, Krzysztof Krajewski, Michael-Christopher Keogh, Mark T. Bedford, Brian D. Strahl

**Affiliations:** 1 Department of Biochemistry and Biophysics, The University of North Carolina, Chapel Hill, NC 27599 USA; 2 Lineberger Comprehensive Cancer Center, The University of North Carolina School of Medicine, Chapel Hill, NC 27599 USA; 3 Curriculum in Genetics and Molecular Biology, The University of North Carolina, Chapel Hill, NC 27599 USA; 4 EpiCypher Inc, Durham, NC 27709 USA; 5 Department of Epigenetics and Molecular Carcinogenesis, The University of Texas MD Anderson Cancer Center, Smithville, TX 78957 USA

**Keywords:** Chromatin, Histone methylation, PHD fingers, Histone peptide microarray, Protein domain microarray

## Abstract

**Background:**

Plant homeodomain (PHD) fingers are central “readers” of histone post-translational modifications (PTMs) with > 100 PHD finger-containing proteins encoded by the human genome. Many of the PHDs studied to date bind to unmodified or methylated states of histone H3 lysine 4 (H3K4). Additionally, many of these domains, and the proteins they are contained in, have crucial roles in the regulation of gene expression and cancer development. Despite this, the majority of PHD fingers have gone uncharacterized; thus, our understanding of how these domains contribute to chromatin biology remains incomplete.

**Results:**

We expressed and screened 123 of the annotated human PHD fingers for their histone binding preferences using reader domain microarrays. A subset (31) of these domains showed strong preference for the H3 N-terminal tail either unmodified or methylated at H3K4. These H3 readers were further characterized by histone peptide microarrays and/or AlphaScreen to comprehensively define their H3 preferences and PTM cross-talk.

**Conclusions:**

The high-throughput approaches utilized in this study establish a compendium of binding information for the PHD reader family with regard to how they engage histone PTMs and uncover several novel reader domain–histone PTM interactions (i.e., PHRF1 and TRIM66). This study highlights the usefulness of high-throughput analyses of histone reader proteins as a means of understanding how chromatin engagement occurs biochemically.

## Background

Histone proteins are fundamental to genome organization and packaging, and are chemically modified by a wide range of “writer” or “eraser” enzymes that, respectively, install or remove histone post-translational modifications (PTMs) [1, 2]. These PTMs play a central role in chromatin function: some are believed to directly impact chromatin organization through biophysical means, but the vast number likely function through their ability to recruit effector or “reader” domain-containing proteins to chromatin. These reader proteins, which are often found in large multi-subunit complexes and in additional chromatin-modifying machines, interact with histone tails and chromatin in various ways that regulate gene transcription and other chromatin functions [2, 3]. The varied and diverse patterns of histone PTMs that exist in vivo are referred to as the ‘histone code’, which is still poorly understood [2, 3].

Histone PTMs often have either activating or repressive effects on gene transcription depending on the type of PTM (acetylation, methylation, etc.) and the position being modified (H3K4, H3S10, etc.). In general, distinct classes of reader domains bind to specific types of PTMs; for example, bromodomains recognize lysine acetylation [4], chromodomains recognize methyl-lysine [5], and the PHD fingers characterized to date generally recognize unmodified or methylated lysine residues [6]. Furthermore, many chromatin-associated proteins contain multiple reader domains, either multiples of the same type [7] or a variety of different domains [8], potentially meaning that the in vivo engagement with chromatin is multivalent. Significantly, increasing evidence shows that dysregulation of the epigenetic machinery, most notably the readers, writers, and erasers of the histone code, is causal for a wide range of human disease, including cancer [9].

Plant homeodomain fingers comprise one of the largest families of reader domains, with over 100 human proteins containing this module [6]. PHD fingers are Zn-coordinating domains that generally recognize unmodified or methylated lysines. To date, the majority of those characterized bind to histone H3 tails either methylated at K4 [7], or unmodified in that position (i.e., KDM5B PHD3 versus KDM5B PHD1 [10, 11] or PHF21A, also known as BHC80 [12]). A smaller number of PHD fingers are reported as readers of H3K9 trimethylation (H3K9me3; e.g., CHD4) [13, 14] and H3K36me3 (e.g., budding yeast Nto1) [15]. Intriguingly, the dual PHD finger region of DPF3b has been reported as a reader of H3K14ac [16], while PHD6 of MLL4 has been reported to recognize H4K16ac [17]. Additionally, a number of these PHD fingers occur in tandem (e.g., MLL1-4 [7] and PZP-containing proteins [18, 19]) or next to additional reader domain types (e.g., bromodomains and chromodomains) [20–22], suggesting combinatorial interaction capabilities.

Despite great progress in uncovering the role of a subset of PHD fingers, many (over 100) of the annotated domain family remain uncharacterized. In this report, we set out to close the gap in our understanding of this reader domain class. Using a combination of complementary approaches (reader domain microarrays, peptide microarrays, pulldowns, and AlphaScreen peptide assays), we show (31/123) of the PHD-containing query proteins to bind histone H3 N-terminal peptides, with the majority of these preferring H3K4me3 over unmodified H3K4. Furthermore, a number of unreported histone PTM–PHD protein interactions were uncovered, with the PHD regions of PHRF1 and TRIM66 binding preferentially to an unmodified H3 N-terminal tail peptide. Given that many of these PHD fingers are mutated in diseases such as breast cancer and leukemia [7, 20–24], these findings enhance our overall understanding of PHD reader–histone interactions and should serve as a resource and platform for future studies.

## Results

### Analysis of the PHD finger proteome via protein domain microarrays

**Fig. 1:**
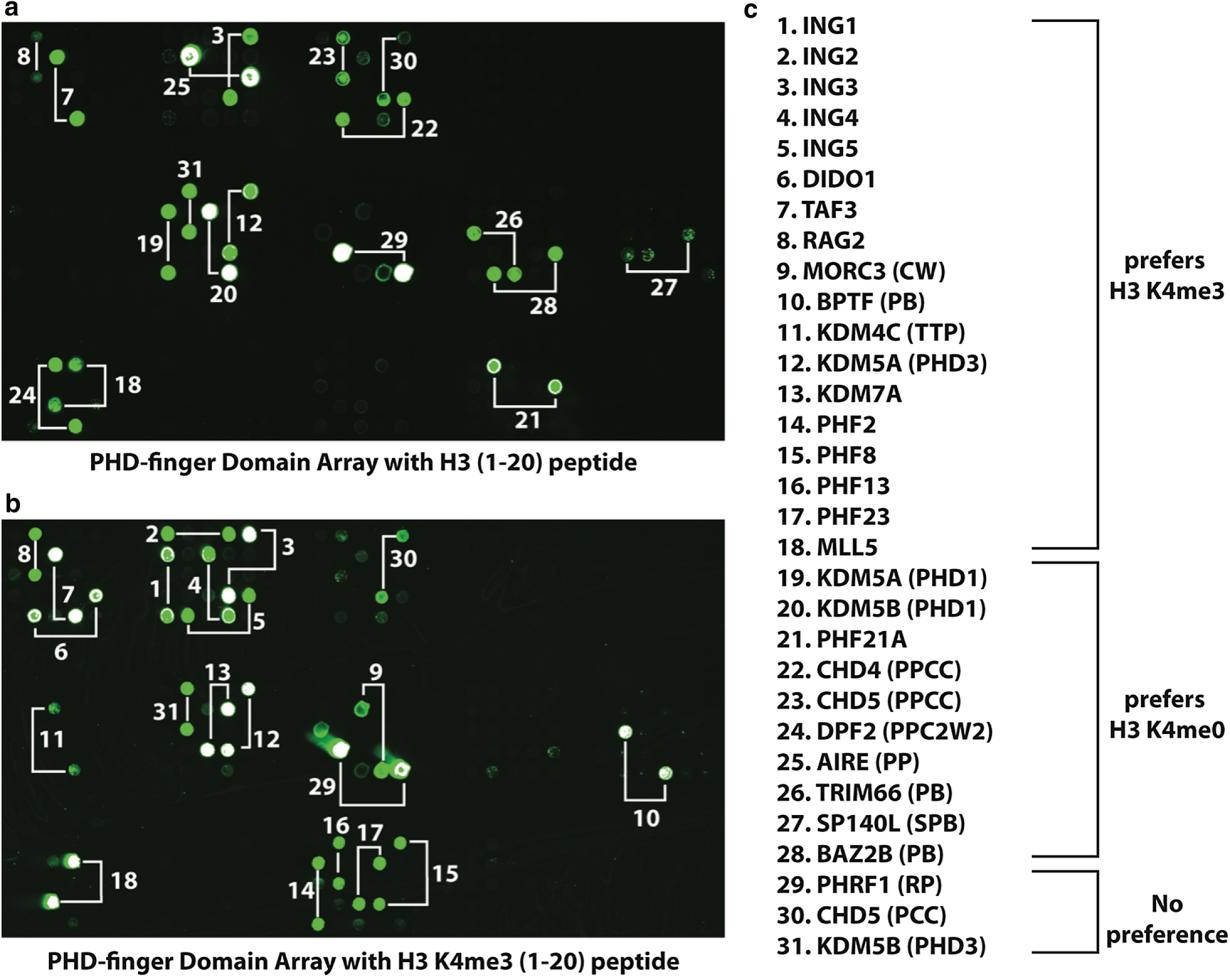
PHD domain array identifies 31 H3-interacting proteins. **a** PHD finger domain microarray probed with an unmodified H3 N-terminal peptide (1–20) (see “Methods”). Each positive binding interaction appears as a green circle, with each PHD protein in the array spotted in technical duplicate (indicated by connecting white lines). **a** PHD finger domain array probed with an H3 (1–20) peptide trimethylated at residue K4 (K4me3). **c** The 31 H3-interacting proteins are listed by their preference for binding H3 (1–20) K4me3 or K4me0. Each protein listed corresponds to the numbers in **a**, **b**. *TTP* Tandem Tudor domain + PHD, *PPCC* Dual PHD + Dual Chromodomain, *PCC* PHD + Dual Chromodomain, *CW* CW-type Zn-finger, *PB* PHD + Bromodomain, *PPC2W2* Dual PHD + C2W2-type Zn-finger, *SPB* SAND + PHD + Bromodomain; domains not indicated, one PHD finger. For the entire list of proteins used and the microarray map, see Additional file 1: Table S1

To define the histone binding preferences of the PHD finger proteome, we expressed and purified 123 annotated human PHD-containing domains as GST-tagged recombinant fusions from *E. coli*. The recombinant proteins consisted of either PHD fingers in isolation, or as tandem domains if a given PHD finger was located adjacent to another reader domain (e.g., one or more PHD fingers, Tudor, chromo and/or bromodomains) (Additional file 1: Table S1). These GST fusions were printed in duplicate on nitrocellulose-coated microarray slides and probed with biotinylated peptides that represented the N-termini of H3, H4, H2A or H2B (Fig. 1a and Additional file 2: Figure S1). As the majority of PHD readers thus far characterized are H3K4me0/3 readers [6], we included additional peptides (H3K4 as either mono-, di-, or trimethylated) to further determine any H3K4 methyl preference (Additional file 2: Figure S2 and Fig. 1b). As a control, we also probed these microarrays with an α-Tubulin peptide (a.a. 30–50) that would not be predicted to interact with PHD fingers (Additional file 2: Figure S1). As in Fig. 1a, b, 31 of the 123 PHD-containing fusions showed positive binding to the H3 N-terminus, with the majority of these interactions showing preference for trimethylated H3K4. In contrast, the H2A, H2B, H4, and tubulin peptides showed little to no positive interactions, suggesting that the PHD finger family broadly prefers the histone H3 tail (Additional file 2: Figure S1). We note that the absence of binding in these experiments does not rule out the possibility of PHD-finger:histone PTM recognition under different hybridization conditions. We also cannot exclude the possibility that some PHD fingers might not be functionally active on the microarrays (perhaps due to misfolding or the lack of an important adjacent region).

Based on the above, we were able to classify the [PHD–H3 tail] interactions into three groups, namely PHD fingers that: (1) bound specifically with methylated H3K4; (2) interacted only with unmethylated H3K4; or (3) bound without preference to the H3K4 methylated state. Many of the PHD fingers found to only bind H3K4 methylation have previously been described and include the well characterized domains from the ING and PHF protein families [6, 24]. The PHD finger of MLL5, a member of the MLL/KMT2 family [25–28], showed strong preference for H3K4me2 and H3K4me3. This finding adds to the relatively small number of MLL5-histone PTM observations reported to date [25]. Of the PHD fingers that bound to H3K4 methylation specifically, we observed that H3K4me3 or H3K4me2 were largely recognized equivalently and these domains did not detect H3K4me1 to the same degree (Additional file 2: Figure S2)—a result in agreement with other reports showing H3K4me binding occurs largely on higher methylated states [6]. Again, as with the H3K4me3 interacting PHDs, our findings for proteins such as KDM5A [third PHD finger (PHD3)] and KDM5B [third PHD finger (PHD3)] are consistent with their current classification as H3K4me3 binders [10, 11]. In contrast to H3K4me2/3 binding, a smaller number of PHD fingers [e.g., PHD1 from KDM5A and KDM5B, PHF21A, AIRE (PP), and TRIM66 (PB)] showed preference for the unmethylated H3K4 state (Fig. 1a, c). Furthermore, three PHD fingers we tested showed no preference between the H3K4me0 and H3K4me3 peptides: PHRF1 (RP), CHD5 (PCC), and KDM5B (PHD3) (Fig. 1). Collectively, these experiments identified 31 PHD-containing reader domains that showed positive interaction with the H3 N-terminus. While a majority of these reader domains preferentially interacted with H3K4me3 (18 out of 31) or H3K4me0 (10 out of 31), three showed no preference for the state of modification at K4. Importantly, these analyses uncovered several reader:histone interactions for poorly characterized PHDs (i.e., TRIM66, PHRF1, and SP140L): such insight could provide new avenues of investigation to these disease-relevant proteins [29–32].

### Further characterization of H3-reading PHD fingers by peptide microarrays

**Fig. 2:**
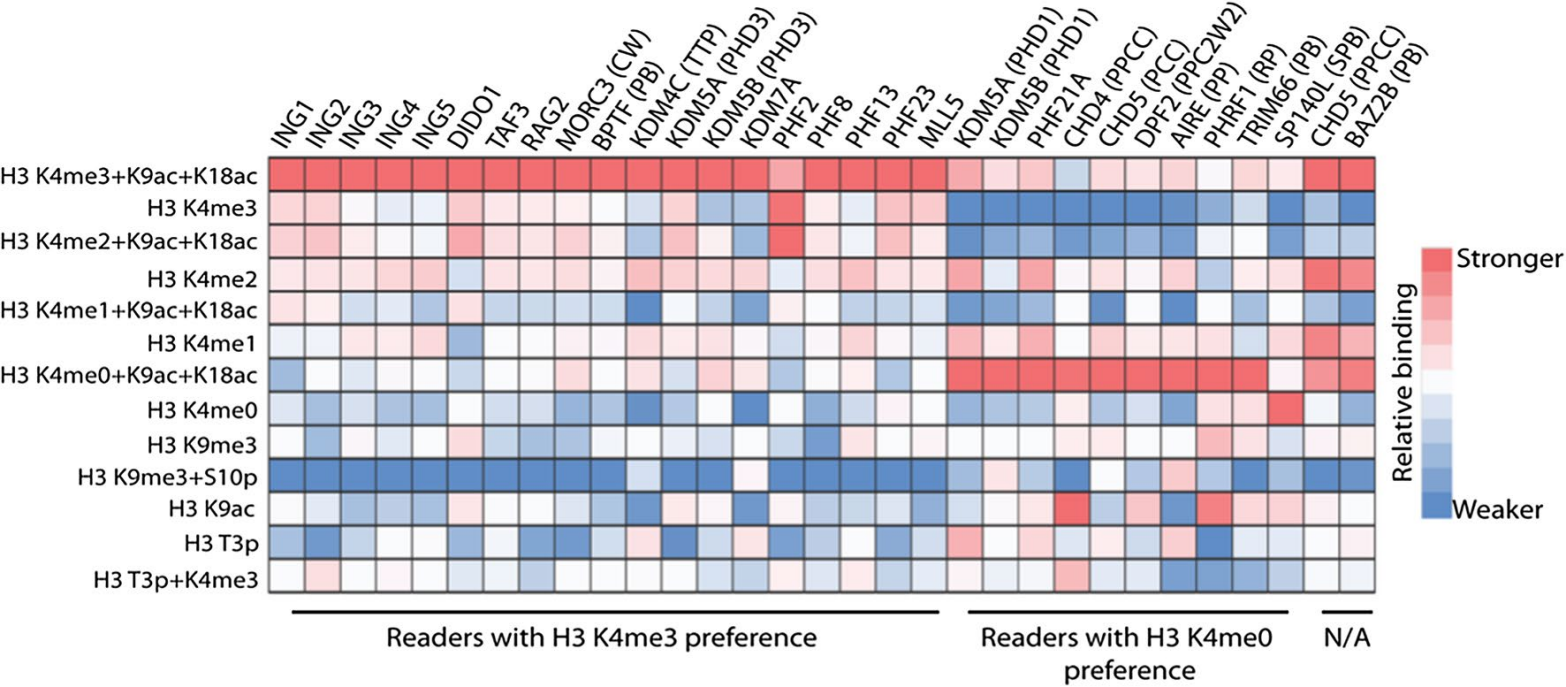
A majority of PHD-containing proteins identified in the domain array are H3 K4me3 readers. The heatmap represents relative binding of the indicated H3 N-terminal peptides (left side) to the PHD-containing GST-tagged proteins (top). Binding strength is shown as a color gradient from red to blue (stronger to weaker). Most of the 31 PHD proteins preferentially recognize H3K4me3 when residues K9 and K18 are acetylated. Array signals (*n* = 4) were normalized individually for each protein to the highest signal for each respective array; thus, comparisons should only be made between binding strengths of different peptides for the same protein. *TTP* Tandem Tudor domain + PHD, *PPCC* Dual PHD + Dual Chromodomain, *PCC* PHD + Dual Chromodomain, *CW* CW-type Zn-finger, *PB* PHD + Bromodomain, *PPC2W2* Dual PHD + C2W2-type Zn-finger, *SPB* SAND + PHD + Bromodomain; domains not indicated, one PHD finger. For full construct information, see Additional file 1: Table S1 and Additional file 2: Figure S3. For full peptide microarray data, see Additional file 3: Table S2

To more comprehensively define the histone interactions of the 31 PHD readers identified from the domain microarray analyses, we probed each on an alternate microarray platform containing a library of 293 synthetic histone peptides with single or combinatorial PTMs [33] (Additional file 2: Figure S4 and Additional file 3: Table S2). All screening results can be found in Additional file 3: Table S2, but for brevity, findings pertaining to peptides that contain K4 and K9 modifications as well as neighboring phosphorylation sites that impinge on the observed binding by reader domains are displayed in the form of a normalized heatmap (Fig. 2). In general, the 31 PHD fingers were confirmed to associate with the H3 tail with the same H3K4 methyl preferences as in the domain microarrays (Fig. 2; Additional file 3: Table S2). Notably, the MLL5 PHD finger displays a strong preference for H3K4me3 over the un-, mono-, or di-methylated H3K4 peptides (Fig. 2), and further, over all other histone peptides on the array (Additional file 3: Table S2), consistent with results from the domain array (Fig. 1). Since CHD4, a protein annotated to recognize H3K9me3 [13, 14], was a positive binder in this assay, we compared its binding to H3K9me3 or H3K4 methyl peptides along with their unmodified counterparts at each position (K4me0/K9me0). The CHD4 (PPCC) fusion bound H3 N-terminal peptides more strongly when H3K4 was unmodified and dually acetylated at K9 and K18 versus when H3K4 is methylated in an identically acetylated context (Fig. 2); additionally, there was no difference in binding to the H3K4me0 peptide versus the H3K9me3 peptide. Interestingly, there also seems to be increased binding with CHD4 (PPCC) to the H3 K9ac peptide, potentially due to the “surface effect” (described in detail below). In addition, we confirmed the newly identified interactions observed with the domain microarrays for PHRF1 and TRIM66 (Fig. 2).

While findings between the domain microarrays and peptide microarrays largely agreed, there were some interesting differences. For example, PHRF1 (RP) showed no preference for the H3K4 methyl state on the domain array but strong preference for H3K4me0 on peptide microarray. Furthermore, KDM5B (PHD3), is reported to bind H3K4me3 [11], and showed such a preference on peptide microarrays but not on domain microarrays (Figs. 1 and 2). It should be noted that the comparison made here is between the H3K4me3 + K9ac + K18ac and the H3K4me0 + K9ac + K18ac peptides. Due to the limited binding, if any, observed by the non-acetylated versions of these peptides, it is difficult to assess the binding preference displayed by KDM5B (PHD3) with this comparison. Of note, certain PHD readers [i.e., DIDO1 and DPF2 (PPC2W2)] also showed some interaction with a number of H4 N-terminal peptides (Additional file 3: Table S2), consistent with published reports [33, 34].

**Fig. 3:**
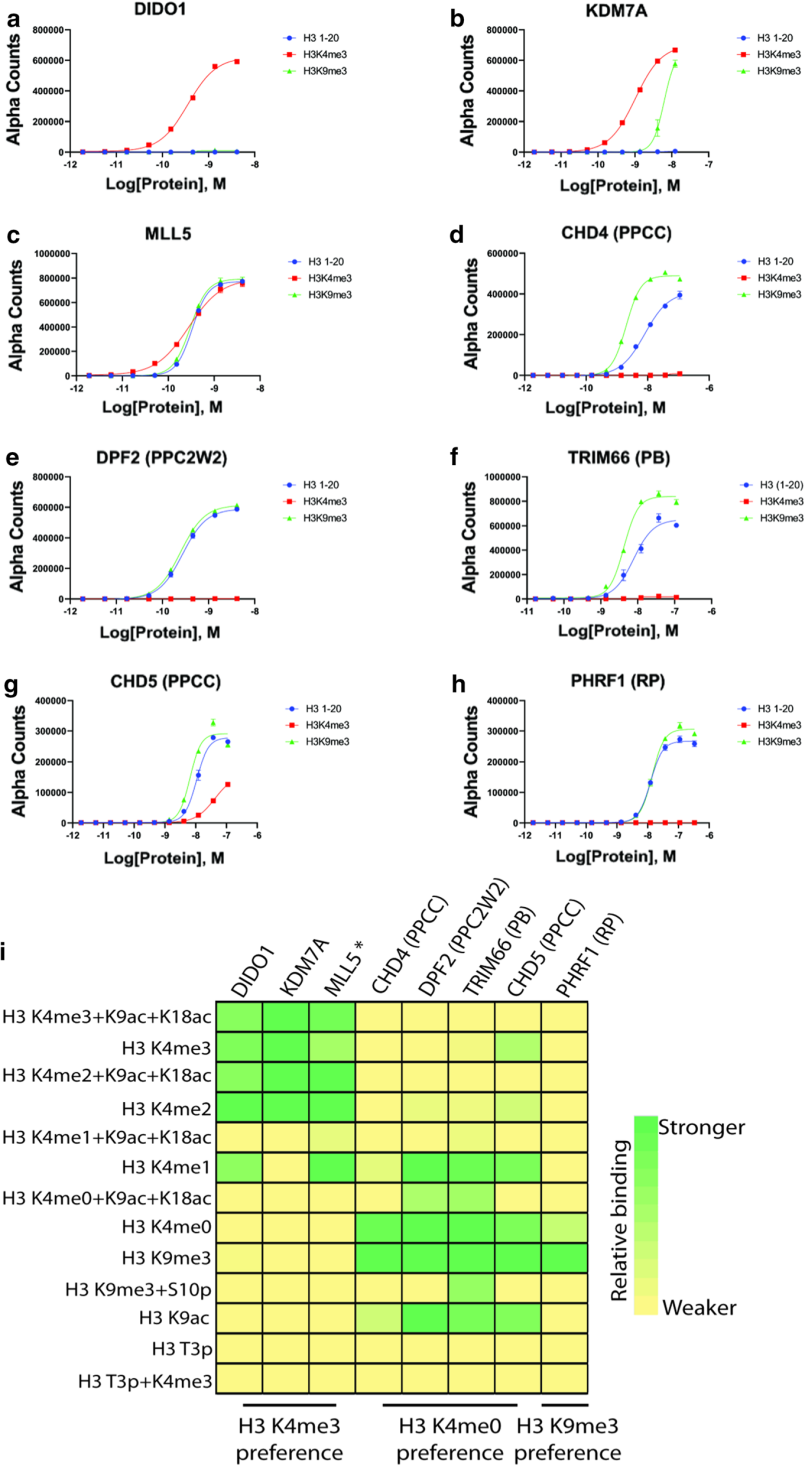
dCypher histone peptide-binding assays define the PTM recognition preference of PHD proteins with high sensitivity. **a**–**h** Binding curves to determine optimal reader protein concentration for full peptide library screening on the dCypher^**®**^ AlphaScreen^**®**^ platform (see “Methods”). *X*-axes are log(protein concentration (*M*)) at constant peptide concentration (100 nM); *Y*-axes are AlphaScreen counts, representing relative strength of binding (*n* = 2; error bars are S.D.). **i** Heat map represents relative binding to H3 N-terminal peptides (left) by PHD-containing GST-tagged proteins (top) using the dCypher AlphaScreen platform. Protein concentrations can be found in Additional file 5: Table S4. Binding strength is indicated by color gradient from green to yellow (stronger to weaker). The asterisk (*) by MLL5 signifies its general preference for H3K4 methylation. Alpha counts (*n* = 2) were normalized individually for each protein to the highest signal for each respective assay. For full dCypher peptide screen data, see Additional file 4: Table S3

During the course of this study, we observed that domain binding to H3 peptides tended to be enhanced when neighboring lysine residues were additionally acetylated (e.g., [K9ac + K18ac] for H3K4me0 or H3K4me readers) (Fig. 2). While at first approximation it might appear that these readers have an enhanced affinity for poly-acetylated states that neighbor H3K4, we note that solution-based peptide pulldown or AlphaScreen (see below) assays with several of these readers (i.e., KDM7A that binds H3K4me3 and KDM5B (PHD1) that reads H3K4me0) did not support this idea (Additional file 2: Figure S5 and Fig. 3i). We surmise that the enhanced binding caused by poly-acetylation is a property of the charged surface of the streptavidin-coated glass slides: when modified with bulky and neutral acetyl groups the highly charged histone N-terminal tail peptides become more accessible to reader domains.

### Quantitative assessment of poorly defined PHD readers by the AlphaScreen dCypher assay

We next employed a highly sensitive proximity-based AlphaScreen histone peptide assay (*dCypher*^®^) to provide a third and orthogonal approach to analyzing the histone binding preferences for a subset of the 31 PHD proteins with respect to various histone tail PTMs. In this assay, biotinylated peptides are bound to streptavidin “donor” beads and the GST-tagged reader domains bound to Glutathione “acceptor” beads. The donor beads are excited by 680 nm light, releasing a singlet oxygen which causes light emission (520–570 nm) in proximal acceptor beads (within 200 nm); emission intensity is then correlated to binding strength [35]. For further examination with this more sensitive approach we chose the PHD fingers with positive binding data from the domain and peptide microarrays that were less characterized in the literature [i.e., MLL5, PHRF1 (RP), and TRIM66 (PB)], or those that displayed weak interactions on the domain and/or peptide microarrays [i.e., CHD4 (PPCC) and CHD5 (PPCC)]. Additionally, we examined several well characterized PHD–PTM interactors [DIDO1, KDM7A, and DPF2 (PPC2W2)] for positive controls and to provide a benchmark. Initial binding assays were conducted for each fusion protein using three peptides [H3 (1–20) with K4me0, H3K4me3 or H3K9me3] to determine the optimal reader domain concentration for full peptide library studies (Fig. 3a–h; Additional file 4: Table S3 and Additional file 5: Table S4). This is an important first step as signal often declined after query protein saturation (the ‘hook point’, caused by excess free query competing with bead bound).

Once the optimal protein concentration ranges for each of the eight readers were determined, we conducted the full dCypher peptide screen (293 histone peptides) (Fig. 3i; Additional file 4: Table S3). In agreement with our previous findings, the dCypher peptide assay demonstrated KDM7A to be a reader of H3K4me3. Furthermore, TRIM66 (PB) showed a preference for H3K4me0 and me1, consistent with findings from the peptide microarrays. For CHD4 (PPCC), the dCypher approach showed a clearer specificity for the H3K4me0 peptide over the methylated species in comparison to the peptide microarray results (Fig. 3i versus Fig. 2). In the case of CHD5 (PPCC), the peptide microarray indicated this protein to be insensitive to the methylation status at H3K4 (Fig. 2), but the dCypher assay identifies a preference for H3K4me0/1 (Fig. 3i), consistent with the domain microarray (Fig. 1a, c).

Consistent with the results from the domain and peptide microarrays, dCypher assays confirmed that the PHD finger of DIDO1 and MLL5 recognized the higher methyl states of K4 (me3/2), but also identified interaction of these domains with the H3K4me1 peptide. Interestingly, the four H3K4me0 readers analyzed—CHD4 (PPCC), DPF2 (PPC2W2), TRIM66 (PB), CHD5 (PPCC)—also showed the ability to bind to the peptides containing H3K9me3; this may be due to H3K4me0 in the H3K9me3 peptide. However, CHD4 (PPCC) and TRIM66 (PB) showed stronger interaction with H3K9me3 compared with the unmodified peptide over a range of protein concentration (Fig. 3d, f). We note that while the initial protein concentration optimizations in Fig. 3a–h were performed over a range of protein concentrations, the full peptide screen (Additional file 5: Table S4; summarized in panel Fig. 3i) was performed at a single protein concentration. When presented with the [H3K9me3 + S10p] peptide, four out of five of the H3K4me0 readers lose binding capacity, suggesting that these readers are sensitive towards the bulky negative phosphate group at S10; this phenomenon is also observed with the H3S10p peptide alone (Additional file 4: Table S3). To our knowledge, this would be the first report of a H3 tail binder outside the H3K9 position to be impacted by S10 phosphorylation, suggesting the phospho-methyl switch may function more broadly than previously thought. Intriguingly, PHRF1 (RP) binding specificity at 15 nM showed more limited interactions to H3K4me0 and H3K9me3 peptides (Fig. 3i), which will be discussed further below. Finally we note that the shift for poly-acetyl peptides seen in the peptide microarrays (reflecting a possible “surface effect”; Fig. 2) is not observed in the dCypher screen (Fig. 3i) which more closely resembles the peptide pulldown assays (Additional file 2: Figure S5).

## Discussion

In the epigenetic landscape, histone PTMs can impact chromatin organization through their ability to recruit effector or “reader” domain-containing proteins. These reader proteins, which are also found in large multi-subunit chromatin-modifying machines, interact with histones and chromatin in various ways that regulate processes from gene transcription to chromosome segregation at mitosis [2]. Given that many of these reader proteins are widely dysregulated in human disease, understanding their histone binding preferences and modes of multivalent interactions is vital [36]. In this study, we screened 123 PHDs (singly and in tandem when next to another reader domain) against the core histone N-terminal tails to dissect the binding preferences for this poorly understood reader domain family. With over 100 PDHs represented on our domain microarrays, we determined that the family strongly prefers the histone H3 tail. Furthermore, the majority of the domains that displayed binding preferred the higher orders of H3K4 methylation, with two subsets showing either a preference for H3K4me0, or no preference to the H3K4 methyl state.

Our findings from domain and peptide microarray confirm the reported binding preferences of many PHD proteins such as those of the ING and PHF families [6, 24]. Additionally, the PHD finger from MLL5 was shown to robustly bind peptides containing each methyl state at H3K4 (me1-2-3) on the domain microarray and dCypher screen, while the peptide microarrays suggest MLL5 is a specific reader for H3K4me3. Intriguingly, we note that previous studies have found discrepancies in whether the PHD finger of MLL5 is a H3K4me3 or H3K4me2 reader [25, 26]. We surmise that the basis of this difference may be due to the overall sensitivity of the various assays employed, which also may account for different observations in the literature. Nonetheless, our analyses provide strong support for MLL5 as a binder of H3K4 methylation on peptides. While recent work has suggested the disease relevance of MLL5 [26], few studies have characterized its histone PTM binding preferences and whether such interaction contributes to its normal or disease functions [25]. The domain microarrays also identified two poorly characterized proteins—TRIM66 and PHRF1—as readers of the unmodified H3 tail. Both proteins are E3 ligases that contain a PHD finger, but whose histone binding capabilities have not been well documented [29–31]. How these histone interactions contribute to the function of these ligases is currently unknown but will be interesting to determine in future studies.

While our domain microarrays revealed 31 out of 123 tested PHD proteins to be binders of the H3 N-terminus (Fig. 1 and Additional file 1: Figures S1, S2), this does not preclude the potential for other PHD fingers to bind under alternate hybridization conditions or to unrepresented targets. Reader domain–histone PTM interactions are multifaceted, and while the results of this study’s domain array do confirm published observations as well as revealing new and interesting binding preferences, we point out that they are not meant to represent an exhaustive list of PHD-mediated interactions but rather to serve as a community resource.

Although domain microarrays are useful in probing many domains in high-throughput, they are limited by the ability to probe with one peptide of interest at a time. To further define the histone PTM landscape to which the subset of 31 PHD proteins identified in the domain microarray might bind, we employed the opposite approach of analyzing each individual domain against a microarray containing ~ 300 singly or combinatorially modified histone peptides (Fig. 2; Additional file 3: Table S2). Through this approach, we were able to confirm many of the interactions observed on the domain microarray with respect to the H3K4me0/1/2/3 peptides. Significantly, the peptide microarray showed that PHRF1 (RP) specifically bound H3K4me0 over K4me, whereas it had no preference on the domain array—which may be explained by the fact that proteins and peptide concentrations on the domain microarrays are high, and thus may capture weak binding events that may not be observed on other platforms.

Despite the obvious potential of peptide microarrays, it would be remiss not to note possible limitations of the platform. The dynamic range of detected interactions is narrow, and from extensive experience, we are only able to characterize domain–peptide interactions on a four-point scale (very strong, strong, weak, or not detected). In addition, these interactions do not represent values that can be translated into binding affinities. Furthermore, comparing values between different probed arrays is also challenging given the lack of a platform control that can be used to normalize signals between arrays. We have also identified potential biophysical artifacts of the platform: we confirmed with these arrays that domains interacting with the H3 N-terminus are influenced by the neighboring acetylation status—a result observed in past publications with PHD readers using these or similar microarrays [37, 38]. However, the impact of H3 acetylation on reader domain binding in the platform context appear to be indirect, as the solution-based binding reactions conclusively show that PHD fingers do not prefer H3K4me0-3 in the context of neighboring acetylation. Rather, it appears that streptavidin-coated slides may carry some amount of negative charge that binds the positively charged histone tails except when this is neutralized (e.g., by acetylation) and thus released from the surface. This “surface effect” shifts the H3 N-terminal binding preferences for many reader proteins towards acetylated peptides, but it is clear that the binding preferences for PHD fingers are primarily driven by direct interactions towards H3K4 (∓ methylation). Although this is a technical challenge, it does not preclude the use of peptide microarrays as the end user can be aware of the role of neighboring acetylation and how to put such results in context.

In contrast to the histone peptide microarrays, the dCypher AlphaScreen histone peptide assay has recently emerged as a highly sensitive and robust technique in gauging the binding interactions between reader domains and histone PTMs [35]. Furthermore, this method allows for the thorough optimization of reaction conditions in terms of buffers, protein/peptide/salt concentration, and cofactor/competitor additives to enable the study of otherwise poorly behaved proteins of interest. Given the advantages of this platform, we used the dCypher assay to first optimize the binding conditions for PHD fingers, and then proceeded to a variety of the PHD fusions that showed low/weak binding or novel histone PTM interactions on the microarrays. The dCypher approach is sensitive and benefits from an initial optimization step for each protein (see Fig. 3a–h) to find the optimal concentration needed in the assay (see Fig. 3i). Using this approach, we were able to confirm that several poorly characterized proteins including TRIM66 are indeed robust readers of H3K4me0 peptides. Intriguingly, the highly sensitive nature of the dCypher assay allowed comparison of peptide-binding signal at low versus high protein concentrations, which revealed that PHRF1 had a distinct binding preference for the H3K9me3 peptide over the H3K4me0 peptide. Importantly, the domain and peptide microarrays rely on micromolar reader domain concentrations, while the dCypher assay can reliably measure binding signal with proteins in the picomolar range. Thus, the dCypher screen revealed the ability of some domains to have distinct preferences at different concentrations that could not be determined from the other approaches. Whether such distinct histone binding preferences in the context of N-terminal peptides are physiologically relevant and could effectively represent the local concentration of particular reader domain on chromatin is currently unknown but is interesting to consider.

## Conclusions

In this report, we have employed multiple high-throughput methods such as domain and peptide microarrays, as well as the proximity-based dCypher peptide screen to assemble a large dataset describing histone PTM binding preferences for PHDs, starting from a broad analysis of the entire family narrowing down to 31 histone H3-interacting readers. While we used the domain microarrays as an initial guide for which proteins to employ in further characterizations, we expect that further exploration of the remaining readers on this microarray platform will uncover additional interactions when binding conditions are further explored (e.g., the PHD domains of UHRF1/2 that were negative in the assays but reported to also bind H3 [39, 40]). Assay development for studying chromatin-interacting proteins has been on the rise in the last decade and we believe that it will be necessary to understand how PHD readers interact with histone PTMs in a nucleosomal context alongside peptides to better replicate physiological conditions. Further, while the bulk of literature and indeed the focus of this study concerning PHD proteins has focused on their interactions with histones, the possibility of these readers binding non-histone biomolecules is intriguing and merits further study. Taken together, we expect our findings to serve as a resource for the chromatin community and to provide a framework for future studies regarding plant homeodomain proteins.

## Methods

### Protein domain array

The protein domain microarray was designed to include 123 GST-tagged PHD-domain containing recombinant proteins. Protein domain microarray development and probing was as previously [41–43]. Briefly, recombinant proteins were synthesized and cloned into pGEX-4T-1 vector by Biomatik Corporation. These GST-PHD readers were subsequently expressed, purified, and spotted in duplicate onto nitrocellulose-coated glass slides (Oncyte Avid slides, Grace Bio-Labs) using a pin arrayer (Aushon 2470, Aushon). For probing, microarray slides were blocked with 3% milk, 3% bovine serum albumin, 0.1% Tween 20 in PBS. Biotinylated peptides were pre-labeled with streptavidin-Cy3 fluorophore (GE Healthcare) and incubated with the blocked array slides. Slides were then washed with PBST and allowed to air dry. Fluorescent interactions were visualized using a GenePix 4200A Microarray Scanner (Molecular Devices).

### Protein purification, histone peptide microarrays, and peptide pulldown assays

The 31 GST-tagged PHD readers identified in the PHD finger domain array were expressed and purified as previously [33]. Histone peptide arrays and peptide pulldown assays were conducted as recently described (specifically, the optimized protocol from Petell et al. for the former) [33].

### dCypher Alphascreen peptide screen assay

The dCypher peptide screen assay was performed as previously described [35]. Briefly, 5 μL of GST-tagged reader domains (optimal protein concentration for library screening determined by initial binding curves to candidate peptides) were incubated with 5 μL of 400 nM (100 nM Final) biotinylated histone peptides (*EpiCypher*) for 30 min at 23 °C in 1× AlphaLISA Epigenetics buffer + epigenetics buffer supplement (*PerkinElmer*, AL1008) in a 384-well plate. A 10 μL mix of 5 µg/mL (2.5 μg/mL final) glutathione Acceptor beads (*PerkinElmer*, AL109M) and 10 μg/mL (5 μg/mL final) streptavidin Donor beads (*PerkinElmer*, 6760002) was prepared in 1× [Epigenetics buffer + supplement] and added to each well. Plates were incubated at 23 °C in subdued lighting for 60 min and AlphaLISA signal measured on a PerkinElmer 2104 EnVision (680 nm laser excitation, 570 nm emission filter ± 50 nm bandwidth).

## Supplementary information

**Additional file 1: Table S1.** PHD finger Domain Array Constructs and Map. *Excel Tab PHD Proteins* is a compiled list of the 123 GST-tagged proteins used for the domain array. The list contains information on the domains, UniProt accession numbers, sequence coverage, and amino acid sequence for each protein. *Excel Tab Array Map* represents the pattern in which each of the GST-tagged proteins has been printed for the domain microarray.

**Additional file 2: Figure S1.** Positive and negative controls of PHD finger domain arrays. **Figure S2.** PHD finger domain array with H3 (1-20) K4me1 and K4me2. **Figure S3.** Domain architecture of the 31 human PHD-containing proteins identified as hits for H3K4me0 or H3K4me3 via protein domain array (Fig. 1). **Figure S4.** Peptide arrays for 31 PHD-containing proteins. **Figure S5.** Peptide Pulldowns with KDM7A and KDM5B (PPC2W2).

**Additional file 3: Table S2.** Histone Peptide Microarray Results. *Excel Tab Peptide List* is a compiled list of the entire 293 histone peptide library with amino acid sequence ranges. *Excel Tab Peptide Array Grid Map* is a table representing the pattern in which peptides have been printed for the peptide microarray. Each number corresponds to the peptide number designated in Peptide List. *Excel Tab Average Signals* is a compiled set of binding data: chromatin reader at 0.5 μM vs. entire 293 peptide library. Data is presented as the average and standard deviation of 4 replicates. The average signals are colored to indicate signal intensity with green being strong and white being weak/low. *Excel Tab Array Heatmap* contains a condensed form of Average Signals data displaying an average of 4 replicates for each chromatin reader tested and individually formatted to demonstrate binding specificity. Key: red= stronger relative binding; blue = weaker relative binding. Excel Tab Array info summary is a table summarizing the domains, sequence coverage, array signal range, and noise for each of the GST-tagged readers assayed on the peptide microarrays.

**Additional file 4: Table S3.** dCypher Results. *Excel Tab Peptide Phase A* is a compiled set of binding data: chromatin reader titration vs. several peptide targets. Data is presented as raw Alpha counts in duplicates at each concentration tested. *Excel Tab Peptide Phase B* is a compiled set of binding data: chromatin reader at optimized concentrations vs. entire 293 peptide library. Data is presented as the average and standard deviation of duplicates. *Excel Tab Peptide Phase B Heatmap* contains a condensed form of Peptide Phase B data displaying an average of duplicates for each chromatin reader tested and individually formatted to demonstrate binding specificity. Key: red = strong binding; blue = weak/no binding.

**Additional file 5: Table S4.** dCypher screen reader domain concentrations.

## Data Availability

The datasets used and/or analyzed during this study are included as additional files. All plasmids are available from the corresponding authors on request.

## Abbreviations

PHD: plant homeodomain
PTM: post-translational modifications
TTP: Tandem Tudor domain + PHD
PPCC: Dual PHD + Dual Chromodomain
PCC: PHD + Dual Chromodomain
CW: CW-type Zn-finger
PB: PHD + Bromodomain
PPC2W2: Dual PHD + C2W2-type Zn-finger
SPB: SAND + PHD + Bromodomain
