# Supplementary material for: Characterization of the plant homeodomain (PHD) reader family for their histone tail interactions
Source: Epigenetics Chromatin. 2020 Jan 24;13:3. doi: 10.1186/s13072-020-0328-z (PMC6979384; doi:10.1186/s13072-020-0328-z)
Supplement: Supplementary file 2 — Additional file 2: Figure S1. Positive and negative controls of PHD finger domain arrays. Figure S2. PHD finger domain array with H3 (1-20) K4me1 and K4me2. Figure S3. Domain architecture of the 31 human PHD-containing proteins identified as hits for H3K4me0 or H3K4me3 via protein domain array (Fig. 1). Figure S4. Peptide arrays for 31 PHD-containing proteins. Figure S5. Peptide Pulldowns with KDM7A and KDM5B (PPC2W2). [file 13072_2020_328_MOESM2_ESM.docx]

**Figure S1.**


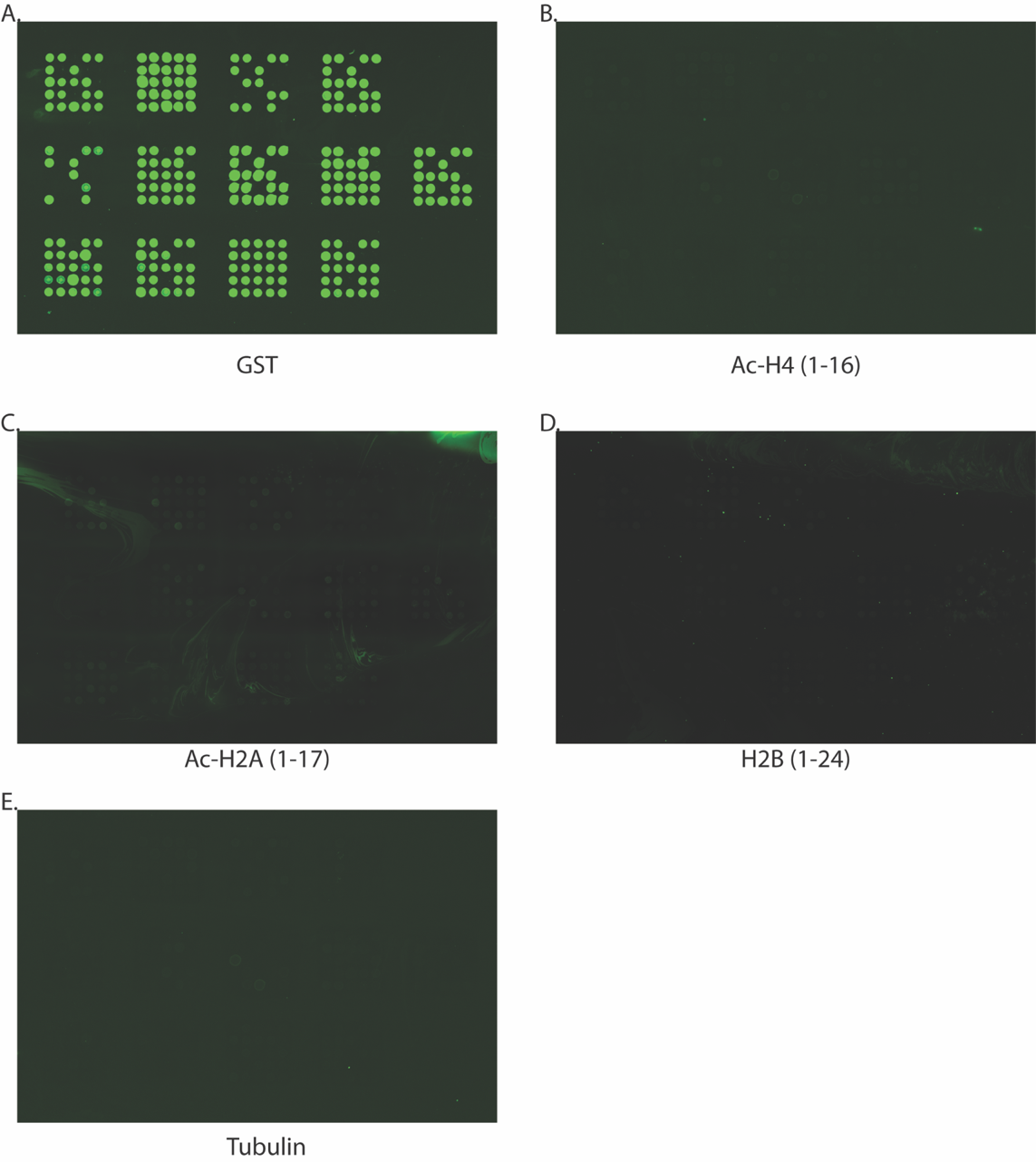


**Figure S1. Positive and negative controls of PHD finger domain arrays.**

**A.** Domain microarray probed for the GST epitope tag on all PHD proteins on the array as a loading / positive control. Each positive binding interaction appears as a green circle, with each PHD protein in the array spotted in technical duplicate. **B.** No detectable binding observed when the microarray is probed with histone peptide Ac-H4 (1-16). **C.** No detectable binding observed when the microarray is probed with histone peptide Ac-H2A (1-17). **D.** No detectable binding observed when the microarray is probed with histone peptide H2B (1-24). **E.** No detectable binding observed when the microarray is probed with an α-Tubulin (30-50) peptide.

**Figure S2.**


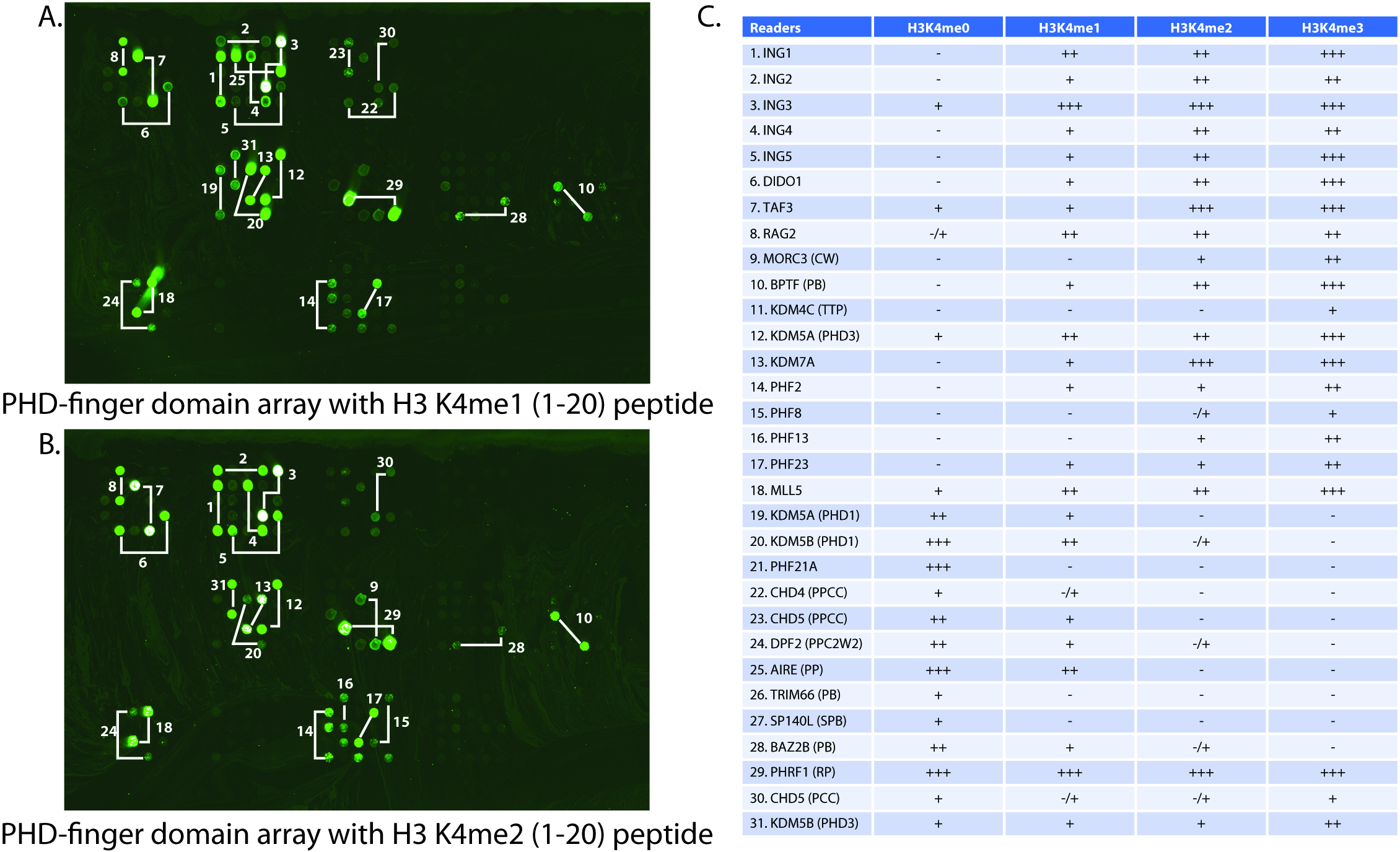


**Figure S2. PHD finger domain array with H3 (1-20) K4me1 and K4me2.** PHD finger domain array probed with an H3 N-terminal peptide from residue 1 to 20 either **A.** monomethylated at K4 or **B.** dimethylated at K4. Each positive binding interaction appears as a green circle, with each PHD protein in the array spotted in technical duplicate (indicated by white lines). **C.** a qualitative summary of binding intensities of the four peptides from Figure 1A, 1B, and panels A and B in this figure. Binding intensities are represented in this table as “-“ (no binding) or from + to +++ (low to high binding). **KEY:** TTP, Tandem Tudor domain + PHD; PPCC, Dual PHD + Dual Chromodomain; PCC, PHD + Dual Chromodomain; CW, CW-type Zn-finger; PB, PHD + Bromodomain; PPC2W2, Dual PHD + C2W2-type Zn-finger; SPB, SAND + PHD + Bromodomain; domains not indicated, one PHD finger. For the entire list of proteins used and the microarray map, see **Supplemental Table S1.**

**Figure S3.**


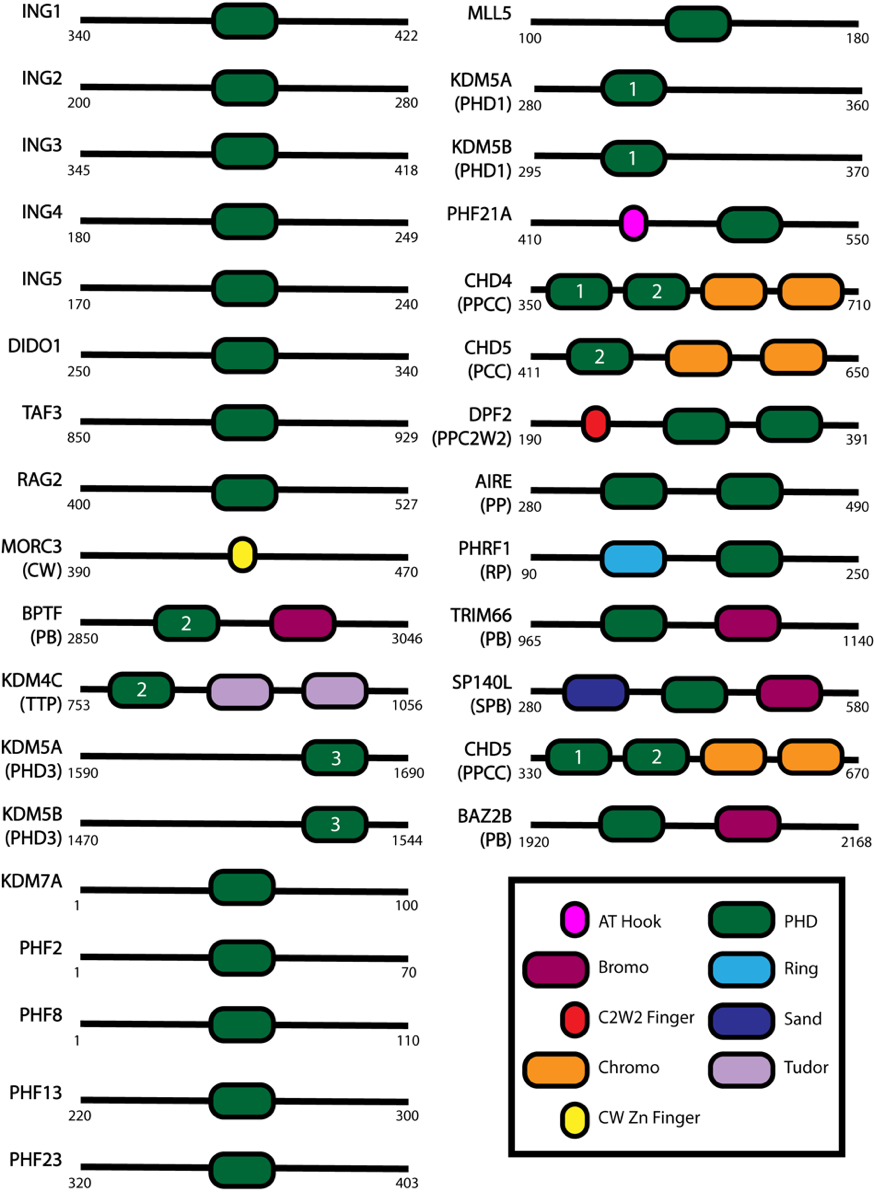


**Figure S3. Domain architecture of the 31 human PHD-containing proteins identified as hits for H3K4me0 or H3K4me3 via protein domain array (Figure 1).**

Constructs with amino acid range spanned, with PHD domains numbered to clarify which are contained within each construct (domains not drawn to scale). AT Hook, DNA minor groove (AT rich) binding domain; Bromo (B), bromodomain; C2W2 Finger (C2W2), C2W2-type zinc-finger domain; Chromo (C), chromatin organization modifier domain; CW Zn Finger, CW- type zinc-finger domain; PHD (P), plant homeodomain; Ring (R), RING- type zinc-finger domain; Sand (S), SAND- type DNA binding domain; Tudor (T), Tudor domain.

**Figure S4.**


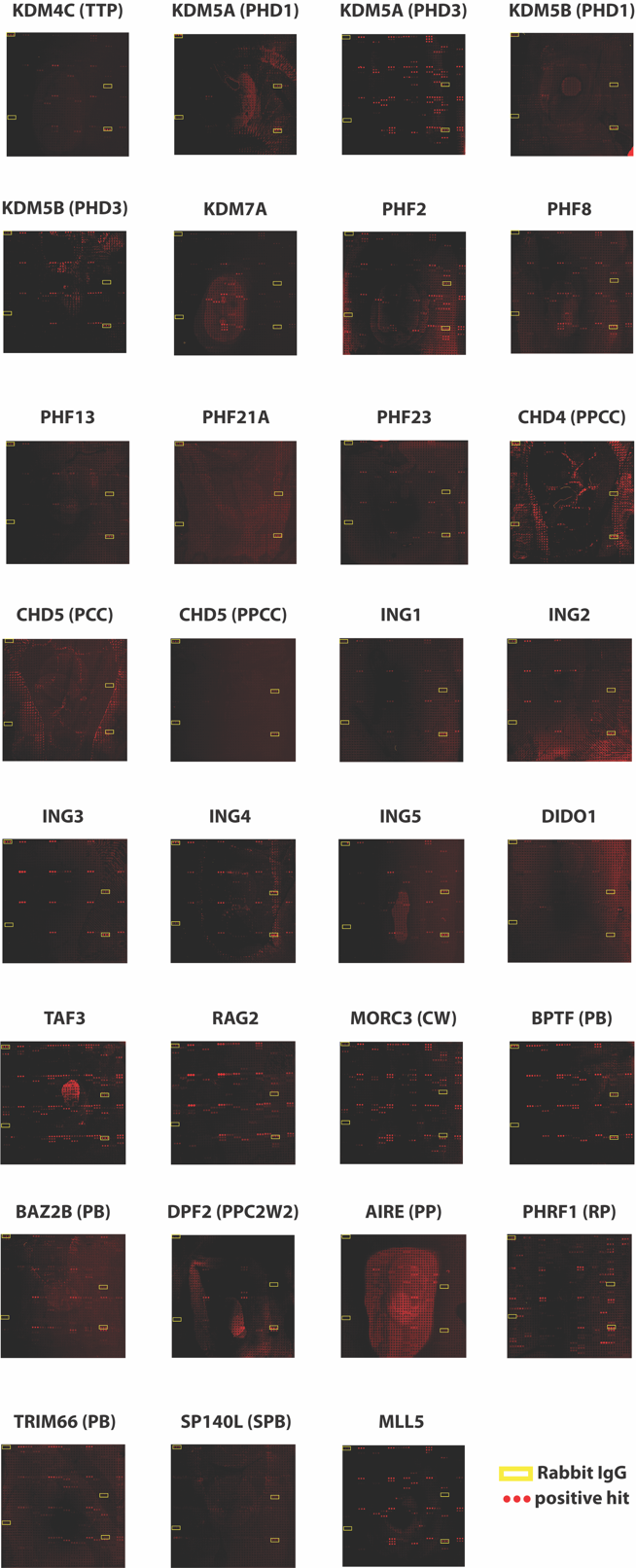


**Figure S4.** **Peptide arrays for 31 PHD-containing proteins.** Each image represents two identical peptide arrays for the indicated GST-tagged PHD-containing protein. A triplet of red circles represents a positive binding event and the rabbit IgG positive controls are boxed in yellow. Each of these arrays were performed twice for a total replicate number (n) of 4. TTP = Tandem Tudor domain + PHD, PPCC = Dual PHD + Dual Chromodomain, PCC = PHD + Dual Chromodomain, CW = CW-type Zn-finger, PB = PHD + Bromodomain, PPC2W2 = Dual PHD + C2W2-type Zn-finger, SPB = SAND + PHD + Bromodomain, and domains not indicated = one PHD finger; for full construct information, see **Supplemental Table S1** and **Supplemental Figure S3**. See **Supplemental Table S2** for information on peptides used in the array and the peptide array map. For full peptide array data, see **Supplemental Table S2**.

**Figure S5.**


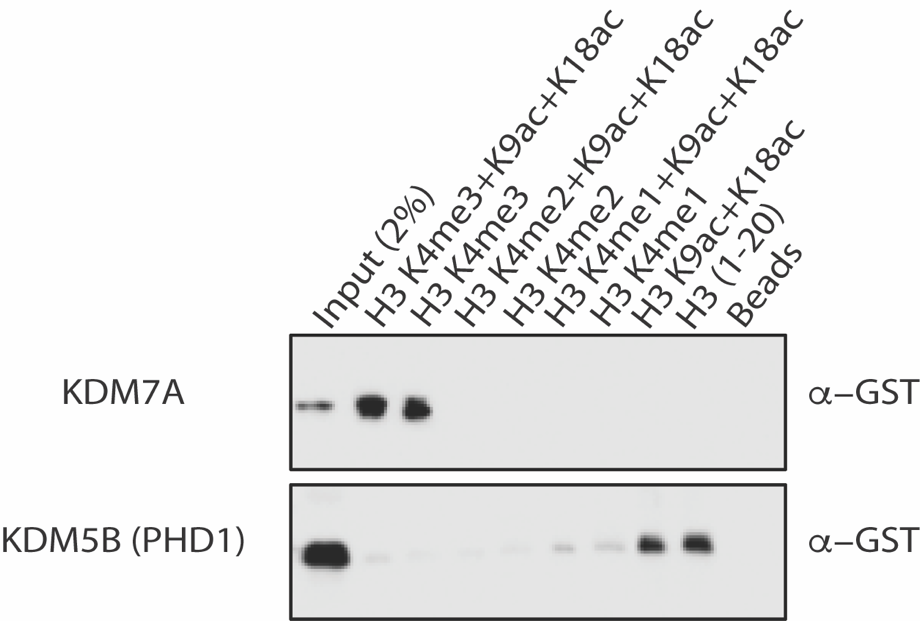


**Figure S5.** **Peptide Pulldowns with KDM7A and KDM5B (PPC2W2).** Representative immunoblot analysis of peptide pulldowns for the PHD fingers of KDM7A and KDM5B (PHD1) with the peptides indicated on the top of the image (n=2).
