# Supplementary material for: Characterization of the plant homeodomain (PHD) reader family for their histone tail interactions
Source: Epigenetics Chromatin. 2020 Jan 24;13:3. doi: 10.1186/s13072-020-0328-z (PMC6979384; doi:10.1186/s13072-020-0328-z)
Supplement: Supplementary file 5 — Additional file 5: Table S4. dCypher screen reader domain concentrations. [file 13072_2020_328_MOESM5_ESM.docx]

**Table S4. dCypher Screen Reader Domain Concentrations**

| **Protein** | **Phase A Optimal Range** | **Phase B Probing Conc.** |
| --- | --- | --- |
| CHD4 (PPCC) | 0.45 – 10 nM | 0.65 nM |
| CHD5 (PPCC) | 1 – 35 nM | 5.2 nM |
| MLL5 | 0.015 – 0.05 nM | 0.02 nM |
| DPF2 (PPC2W2) | 0.05 – 1 nM | 0.1 nM |
| KDM7A | 0.05 – 1 nM | 0.15 nM |
| DIDO1 | 0.015 – 0.5 nM | 0.1 nM |
| PHRF1 (RP) | 5 – 30 nM | 15 nM |
| TRIM66 (PB) | 0.5 – 10 nM | 7 nM |
